# Computational flow cytometric analysis to detect epidermal subpopulations in human skin

**DOI:** 10.1186/s12938-021-00858-8

**Published:** 2021-02-17

**Authors:** Lidan Zhang, Ying Cen, Qiaorong Huang, Huifang Li, Xianming Mo, Wentong Meng, Junjie Chen

**Affiliations:** 1 Department of Burn and Plastic Surgery, West China Hospital, Sichuan University, Chengdu, 610041 Sichuan China; 2 Laboratory of Stem Cell Biology, State Key Laboratory of Biotherapy, West China Hospital, Sichuan University, Chengdu, 610041 Sichuan China

**Keywords:** Flow cytometry, Heterogeneity, Human epidermis, Machine learning

## Abstract

**Background:**

The detection and dissection of epidermal subgroups could lead to an improved understanding of skin homeostasis and wound healing. Flow cytometric analysis provides an effective method to detect the surface markers of epidermal cells while producing high-dimensional data files.

**Methods:**

A 9-color flow cytometric panel was optimized to reveal the heterogeneous subgroups in the epidermis of human skin. The subsets of epidermal cells were characterized using automated methods based on dimensional reduction approaches (viSNE) and clustering with Spanning-tree Progression Analysis of Density-normalized Events (SPADE).

**Results:**

The manual analysis revealed differences in epidermal distribution between body sites based on a series biaxial gating starting with the expression of CD49f and CD29. The computational analysis divided the whole epidermal cell population into 25 clusters according to the surface marker phenotype with SPADE. This automatic analysis delineated the differences between body sites. The consistency of the results was confirmed with PhenoGraph.

**Conclusion:**

A multicolor flow cytometry panel with a streamlined computational analysis pipeline is a feasible approach to delineate the heterogeneity of the epidermis in human skin.

## Background

As the outermost layer of the skin, the epidermis provides an effective barrier between the human body and the environment [1]. The epidermis is generally composed of the interfollicular epidermis (IFE) and adnexal structures, including the hair follicles (HFs), pilosebaceous units (PSUs), and sweat glands [2]. The IFE contains 4 spatial layers: the stratum basale (SB), the stratum spinosum (SS), the stratum granulosum (SG), and the stratum corneum (SC). The permanent part of HF is divided into anatomically and biochemically distinct compartments, including the infundibulum, junctional zone, isthmus, bulge, and hair germ (HG) [2].

The epidermis regenerates itself throughout life constantly as the body’s most dynamic tissues [3]. The epidermal stem cell populations are lineage restricted within their own respective territory during homeostasis with the capacity to differentiate into all epidermal lineages [4, 5]. The basal cell layer of IFE remains in contact with the dermis through integrin-based adhesion and hemidesmosomes and express basal marker proteins, such as keratin 5 (K5) and K14 [3, 6]. The expression of CD49f and CD29 in the basal IFE is confined constitutively in human epidermis, both of which are putative markers of IFE stem cells [3, 7]. There is increasing evidence that IFE stem cells are heterogeneous [8, 9]. The rete ridge pattern is a particular feature providing mechanical strength to the skin and improving the nutrient supply to the avascular epidermis by increasing the surface area of epidermal–dermal junction as well as the capillary–epidermal interface [10]. The tip of the rete ridge suggests the enrichment of epidermal stem cells in rete ridges with high expression of keratin 15(K15) and CD 117 [7, 10, 11]. Once the differentiation begins, basal cells become detached from the basement membrane, reorganize adhesive junctions and cytoskeleton, case expression of the basal markers and start terminal differentiation programs with expression of differentiation markers, including K1 and K10, early-stage differentiation marker, Loricrin and Filaggrin, late-stage differentiation marker [3, 5, 6]. CD24 is a small and highly glycosylated protein expressing in the suprabasal layer and stratum spinosum, rarely in stratum granulosum [7]. HFs are composed of 3 distinct epithelial layers: the outer root sheath (ORS), the inner root sheath (IRS) and the hair shaft [12]. Heterogeneous stem cell populations can be observed in both hair germs and bulges [13, 14]. K15 and CD34 are expressed in the ORS as a stem cell marker, while the expression of cytokeratin 16 (K15) is detected in all HFs [7, 12].

The study of epidermal heterogeneity could lead to an improved understanding of the renewal cycle during homeostasis, wound healing, and disease status, such as tumor progression and skin aging, as well as the identification of stem cells in the epidermis [6]. Additionally, the application of epidermal stem cells plays a crucial role in the field of regenerative medicine [15, 16]. Clear identification of the epidermal cell population, as the basis of the field, has been a compelling and inconclusive topic due to the lack of specific biomarkers [6]. Given the importance of epidermal heterogeneity, the cell subpopulations in the human epidermis urgently need to be clarified.

Flow cytometry has been proven to be an efficient and reliable method to identify and sort epidermal cells. As the application of flow cytometry has played a critical role in the search for specific markers of human epidermal subgroups and in sorting of these cells for further studies, an optimized multicolor flow cytometry (MFC) panel is urgently needed [17–19]. Here, we developed and optimized a 9-color panel to detect distinct subgroups of human epidermal cells. To accurately visualize heterogeneity, visualized t-distributed stochastic neighbor embedding (viSNE) was applied to reduce dimensionality, which preserved high-dimensional proximity relationships [20, 21]. The heterogeneity among human epidermal cells of different ages and from different locations was demonstrated by clustering with the PhenoGraph and Spanning-tree Progression Analysis of Density-normalized Events (SPADE) algorithms to compare the effectiveness of manual and computational analysis [20, 22].

## Results

### Manual flow cytometry analysis revealed the heterogeneity of epidermal cells

**Fig. 1:**
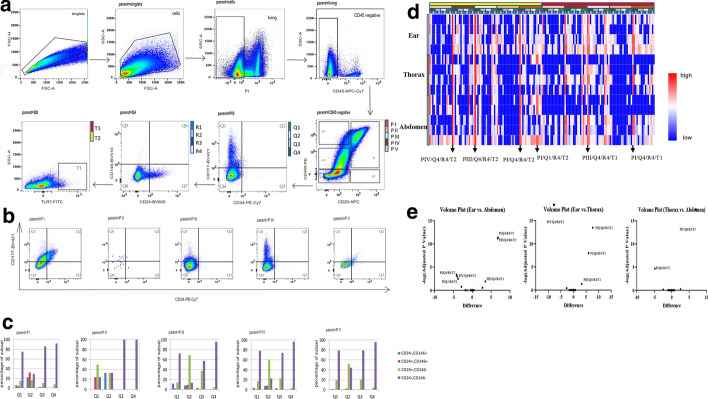
A representative sample shows the subgroup phenotype by manual analysis. **a** The manual gating strategy for the panel. This strategy removes debris, doublets, low viability cells, and CD45-positive cells. The cellular phenotype was displayed in a serial biaxial gates. **b**, **c** The phenotypes of PI to PV. **d** The heatmap of each subset percentage of live epidermal cells. The first four lines indicated the immunophenotype shown in B. Left row indicated the body sites as ear, thorax and abdomen. Logarithmic conversion of percentage values was performed. **e** Volcano plot of subsets which have statistical difference between body sites

After excluding debris, dead cells and CD45-positive cells, the whole epidermal cell population was analyzed with a sequence of manual biaxial gates, which divided the group into five subgroups, generally PI–PV based on the expression of CD49f and CD29 (Fig. 1a). The heterogonous phenotype of each subgroup was demonstrated by the expression of CD117, CD34, CD146, CD24 and TLR7 (Fig. 1b, c). In total, the whole epidermal cell population was divided into 160 subpopulations. PI and PV shared a similar pattern in expression of CD 117 and CD 34 with an obviously co-positive subpopulation, while PIII and PIV demonstrated a CD117-positive subpopulation (Fig. 1b). In the further analysis, the expression of CD146 and CD24 demonstrated the phenotypic variation in each subgroup (Fig. 1c). The CD 117 + /CD34 + subgroup in PV composed with CD24 + /CD146- and CD24-/CD146- cells generally, while the other co-positive subgroup was composed with several distinct subsets (Fig. 1c). The subpopulations displayed some differences among the ear, thorax and abdomen, with a tendency toward more TLR7^+^ subgroups in the ear and more TLR7^−^ groups in the abdomen (Fig. 1d). The percentage of CD49f^hi^/CD29^+^/TLR7^+^ and TLR7^+^ subpopulations was statistically higher in the ear, compared with the thorax and abdomen (Fig. 1e). At the same time, the percentage of CD49f^med^/CD29^−^ subpopulation decreased in the ear (Fig. 1e).

### Automatic clustering reveals different proportions in the ear, thorax and abdomen

**Fig. 2:**
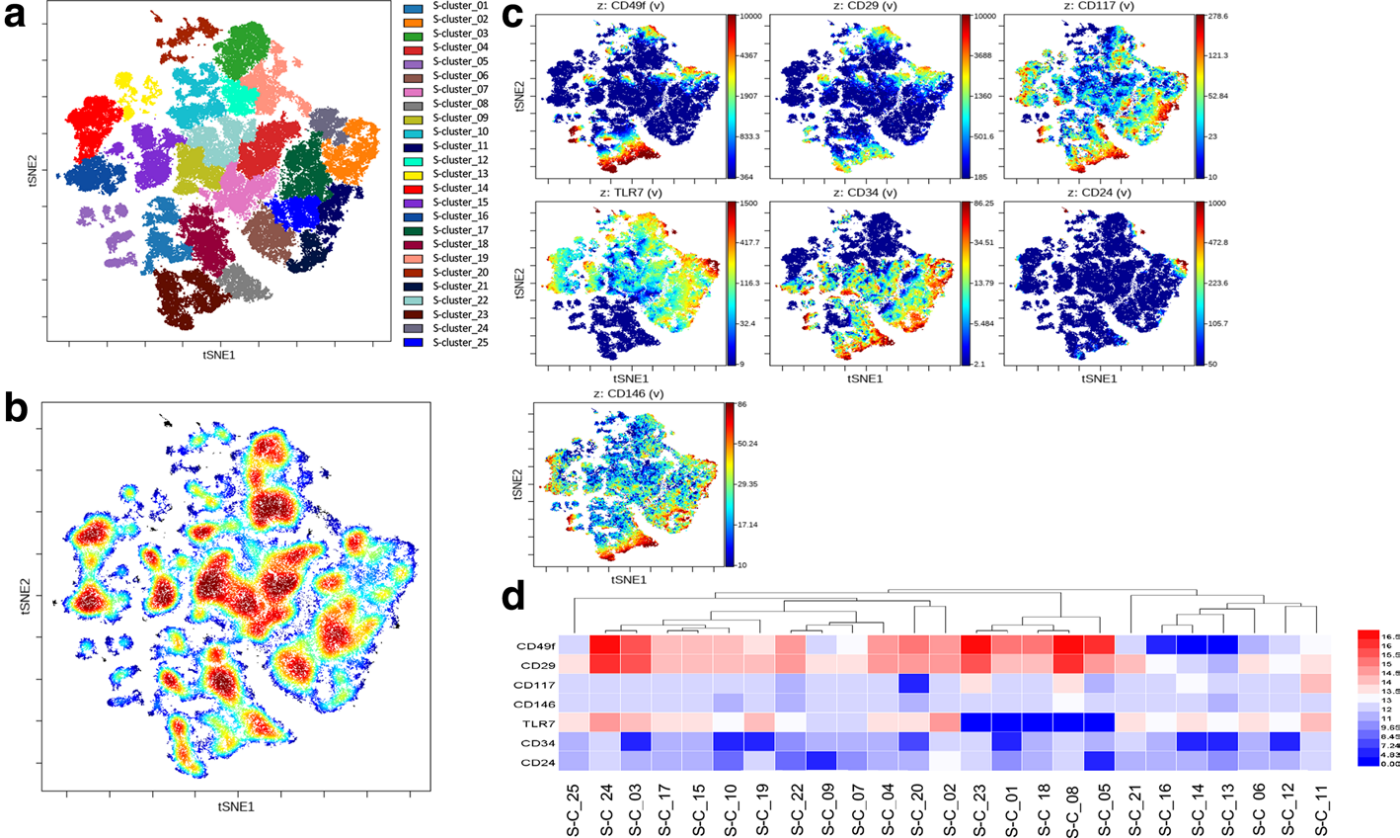
viSNE analysis on the epidermal cells imported from live CD45-negative single cells, concatenated data of all the normal samples (*n *= 14). **a** viSNE maps define 25 spatially distinct cell clusters. viSNE map showing the cell density of all concatenated cells. **b** viSNE map, colored by the cell density of all concatenated events. **c** cells from the whole epidermis, colored by the expression of CD49f, CD29, CD117, TLR7, CD34, CD24 and CD146. **d** Mean fluorescence intensity of surface markers for each cluster calculated Arcsinh ratio of means controlled by table's minimum

In this study, the SPADE algorithm clustered the epidermal cell population into 25 clusters based on viSNE visualizing the high-dimensional data on a biaxial map in terms of tSNE-1 and-2 (Fig. 2a). ViSNE plotted each cell according to high-dimensional distance so that it conserved the cluster similarity and structure. Several low-abundance cell islands were clustered into the same cluster to simplify further analysis automatedly (Fig. 2b). Although most of the phenotypic markers displayed in gradients of expression, CD24 showed a clustered expression (Fig. 2 c). The general low expression of CD24 may be related to the fact that the stratified cells were mostly excluded as dead cells. Within the subpopulation of cells that highly expressed CD49f, the expression of CD29 was also highly expressed (Fig. 2d).

**Fig. 3:**
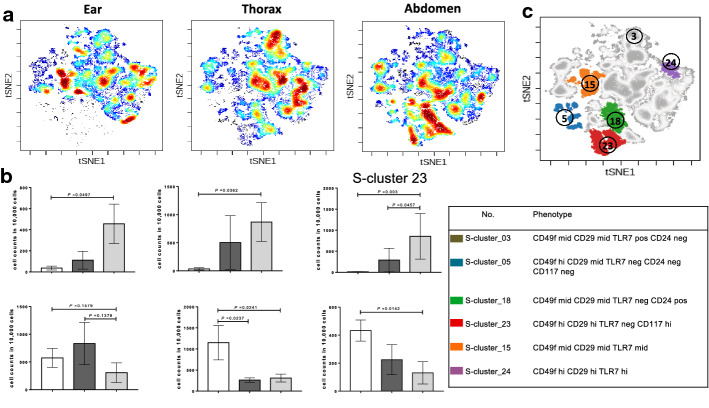
viSNE reveals different distribution in ear, thorax and abdomen. **a** viSNE map concatenated by ear, thorax and abdomen. **b** Cell account in S-cluster 05, 18, 23, 03, 15 and 24. **c** Overlaid viSNE map of S-cluster 05, 15, 18, 03, 23 and 24 demonstrating the spatial locations. Data are plotted as mean ± SEM

**Fig. 4:**
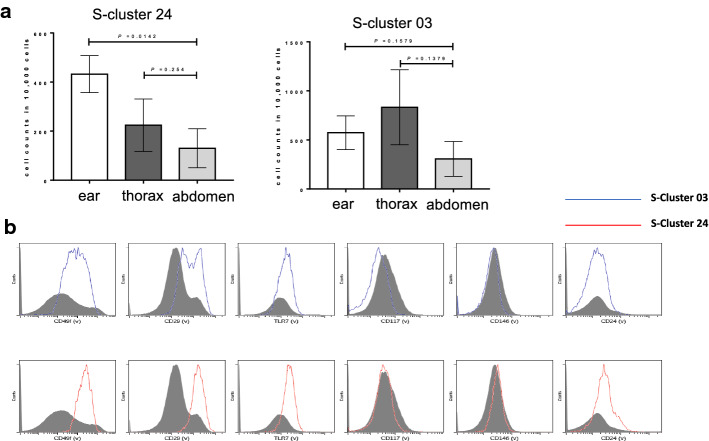
S-cluster 03, 24 abundance in the ear, the thorax and the abdomen.** a** cell counts in cluster 24 and 03 in ear (*n* = 4), thorax (*n* = 5) and abdomen (*n* = 5) showing significant difference between the ear and thorax, thorax and abdomen. Data are plotted as mean ± SEM. **b **fluoresce intensity of CD49f, CD29, TLR7, CD117, CD24 and CD146 in S-cluster 03 and 24

To compare the phenotypic character of epidermis from different body sites, we concatenated the fcs files by the ear, thorax and abdomen. The viSNE map of the ear, thorax and abdomen showed different distributions of epidermal cells (Fig. 3a). In the abdomen, S-cluster_5 (CD49f^hi^/CD29^mid^/TLR7^neg^/CD24^neg^), 18 (CD49f ^mid^/CD29^mid^/TLR7^neg^/CD24^pos^), and 23 (CD49f^hi^/CD29^hi^/TLR7^neg^/CD117^hi^) were 10.62-, 13.19-, and 24.23-fold higher in the ear, while S-clusters 15 (CD49f ^mid^/CD29^mid^/TLR7^mid^) and 24 (CD49f^hi^/CD29^hi^/TLR7^hi^) were 3.70- and 3.32-fold lower in the ear, respectively (Fig. 3b). The heat map of mean fluorescence intensity (MFI) showed that S-cluster_03 (CD49f^hi^/CD29^hi^/TLR7 ^hi^/CD24^−^/CD117^−^/CD146^−^) and 24 (CD49f^hi^/CD29^hi^/TLR7^hi^/CD24^+^/CD117^−^/CD146^+^) shared similar expression patterns (CD49f^hi^/CD29^hi^/TLR7^hi^) (Fig. 2d). The location of S-cluster_05, 18 and 23 gathered at the left corner, while S-cluster_15 and 24 separated at two sides of the tSNE map (Fig. 3c). Statistical analysis demonstrated that the percentage of S-cluster_03 was highest (5.74%) in the ear and lowest (3.07%) in the abdomen (*p* < 0.05), while the percentage of S-cluster_24 was highest (4.32%) in the ear (thorax: 2.24%, abdomen: 1.30%) (Fig. 4a). The phenotypic character of S-cluster_03 and _24 was shown in histogram (Fig. 4b).

### PhenoGraph shows clustering consistent with SPADE on viSNE result

**Fig. 5:**
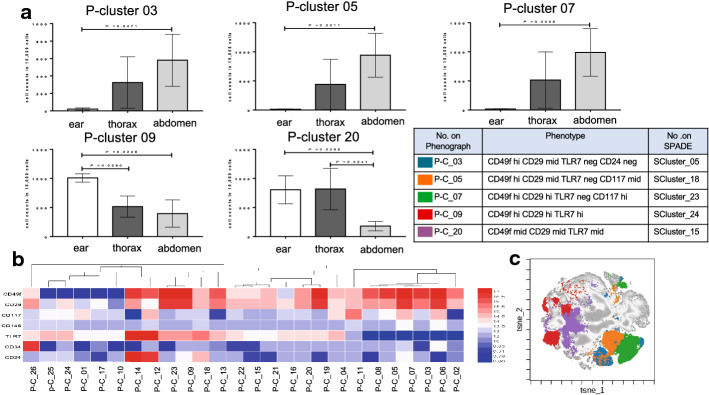
Phenograph analysis of epidermal cell in the ear, thorax and abdomen. **a** cell account in P-cluster 03, 05, 07, 09 and 20. **b **MFI of each cluster demonstrating the phenotype. **c** Overlaid viSNE map of P-cluster 03, 05, 07, 09 and 20 demonstrating the spatial locations. Data are plotted as mean ± SEM

To validate the reliability of SPADE on viSNE, the initial fcs files and concatenated files were uploaded and ran in PhenoGraph (10,000/sample, iterations: 7000, perplexity: 50, seed: 42, k: 45). P-cluster_03, 05, 07, 09 and 20 demonstrated significant differences between groups (Fig. 5). P-cluster_03 (CD49f^hi^/CD29^mid/^TLR7^neg^/CD24^neg^), 05 (CD49f^hi^/CD29^mid^/TLR7^neg^/CD117^mid^), and 07 (CD49f^hi^/CD29 ^hi^/TLR7^neg^/CD117^hi^) were highest in the abdomen (cluster_03: 5.81%, 05: 9.42%, and 07: 9.90%). P-cluster_09 (CD49f^hi^/CD29^hi^/TLR7^hi^) and 20 (CD49f^mid^/CD29^mid^/TLR7^mid^) were highest in the ear (cluster_09: 10.01%, 20: 8.06%). The cluster number in PhenoGraph and SPADE was not in one-to-one correspondence. The cluster number was corresponding in the basis of the phenotypic character (Fig. 5).

## Discussion

The heterogeneity of the epidermis reflects cell development and location. The IFE maintains homeostasis by moving the SB cells upwards to form the SS, SG, and SC [8, 23]. The HFs renew from the bulge area. While differentiating, the state and function of the epidermal cells change as do their surface markers. Additionally, heterogeneous stem cell populations have been proven to be present in the basal layer and HFs [24]. Recent studies have indicated the existence of various stem cell populations that maintain their proliferative frequency under steady-state condition in the IFE and HFs [6, 25]. MFC provides an effective and simple tool to detect and sort epidermal cells for downstream application [19, 26, 27]. A valid polychromatic flow cytometric panel could be a useful tool for identifying various populations of epidermal cells.

This panel was developed with specific criteria and guidelines. The primary considerations throughout the process were epitope expression levels, fluorescein brightness, and commercial clone availability. As integrin plays a fundamental role in keratinocyte adhesion and migration, a high expression level of CD49f and CD29 indicates proliferative and transit-amplifying cells in the basal layer and bulge [28]. CD117, a rete ridge marker, is enriched on a mixture of dormant stem cells, melanoblasts, and melanoma cells without expression at the bulge [7, 29]. In healthy adult skin, CD146 is expressed at the epidermal appendage and the external root sheath of HFs [30, 31]. CD24 is detected in postmitotic, nonclonogenic suprabasal keratinocytes located in the stratum spinosum of the IFE and the outer and inner root sheath of HFs [32, 33]. CD24 has been identified as a differentiation marker of keratinocytes [34]. CD34 is a heavily glycosylated 110-kDa transmembrane protein. CD34^+^ epithelial cells have been confirmed to be present at the outer root sheath of anagenic human HFs [35]. TLR7 is an intracellular Toll-like receptor that is known for its importance in autoimmunity [36, 37]. Recently, TLR7 has been proven to be a surface marker for skin stem cells in mice [38]. In this panel, the antibody cocktail combination was developed to dissect the IFE layer by layer and subsets of these layers, as well as subsets in HFs, which could provide inspiration for studying the structure of the human epidermis. Given the epidermis is a closely arranged barrier, the trypsinization effect was estimated with H&E staining, which showed the epidermal cells detached after enzyme digestion (Additional file 1: Fig. S1).

Manual gating in flow cytometric analysis uses biaxial plots to display the expression of multiple markers with the risk of overlooking small changes [21]. In our panel, the manual gating strategy was performed by an experienced expert, with the epidermal cells divided into five subgroups based on CD49f and CD29 expression. Charruyer et al. revealed that there was no significant alteration of the epidermal stem cell frequency during aging through long-term repopulation in vivo and colony formation in vitro, and we found that the frequency of CD49f^hi^/CD29^+^ subpopulation between kids and adults showed no difference in flow cytometric analysis (data not shown)[39]. The MFC was used to delineate the phenotypic signatures among body sites. The distribution of subgroups among the ear, thorax and abdomen indicated a tendency toward more TLR7^+^ subsets in the ear, especially the TLR7^+^ group in PI. CD49f^hi^/CD29^+^ subgroups indicated a proliferative subset of basal epidermal cells. Yin et al. demonstrated the stemness of TLR7^+^ cells. The CD49f^hi^/CD29^+^/TLR7^+^ subset indicated one or more epidermal stem cell subpopulations [38]. The decrease of CD49fmed/CD29- subpopulation, which presented a differentiated subgroup of keratinocytes, was consistent with elevating portion of more proliferative subsets in the IFE.

Computational analysis makes it possible to cluster epidermal subsets in an unbiased way and reveals the possibilities for cellular identification in future clinical assessment by dimensional reduction and machine learning algorithms, which relies less on the experience of the operators [40]. viSNE is a common used algorithm for high-dimensional data with a limitation of crowding problem, which could be solved with the combination of clustering method, such as SPADE [41]. There were 25 clusters confirmed by SPADE on viSNE, which decreased the workload to determine the differences between groups compared to manual analysis, which divided the whole epidermal cells into 160 subpopulations. The cluster distributions of the ear, abdomen, and thorax demonstrated distinctive patterns (Fig. 3a). In further quantitative analysis of the cluster cell count, significant differences were confirmed (*p* < 0.05) by multiple t-tests for each cluster. S-clusters_15 and _24 expressed the highest levels in the ear, while S-clusters_05, _18 and _23 expressed the highest levels in the abdomen. The phenotype of each cluster was confirmed with the MFI heatmap, demonstrating negative expression of TLR7 in S-cluster_05, 18 and 23 and positive expression in S-cluster_15 and 24. Considering that Yin et al. reported TLR7 as an epidermal stem cell marker, the distinct expression pattern of TLR7 indicates the possibility that TLR7 could be a stem cell surface marker in the human epidermis [38]. The phenotype consisting of S-cluster_05, 18 and 23 was TLR7^−^/CD24^+^, which indicated differentiated epidermal cells. At the same time, the phenotype of S-cluster_15 and 24 indicated possible stem cells, with a CD49f^hi^/CD29^+^/TLR7^+^ phenotype. Variation in the epidermis at different body sites has been reported with various methods, including reflectance confocal microscopy and flow cytometry [42, 43]. Webb et al. sorted human skin epidermis labeled with CD49f, CD71 and 14, K10, and K15, analyzed the cell cycle of each subset and found that epidermal stem cells (CD49f^hi^/CD7^neg^) were decreased in the sun-exposed area [43]. Our results demonstrated that a lower frequency of the CD49f^hi^/CD29^+^ subpopulation in the ear than in the chest and abdomen, which are sun-exposed areas.

Several computational clustering algorithms have been established for MFC or mass cytometry, of which SPADE and PhenoGraph are commonly applied to stratify all events into subpopulations [44]. The PhenoGraph analysis divided the whole epidermis into 26 clusters, of which 5 clusters demonstrated significant differences. The phenotypes and percentages of these clusters revealed that these clusters are the same in SPADE analysis, which indicates the consistency of computational analysis. Compared to manual analysis, a supervised method, machine learning, as an unsupervised clustering method, gives a holistic and clear view of the whole panel.

## Conclusion

In this study, we established a polychromatic flow cytometry panel to delineate the human epidermal cell phenotype characteristics and visualize the high-dimensional data by t-SNE/SPADE, an automatic data analysis approach to identify different human epidermal cell subsets in an unbiased way.

## Methods

Aseptic samples of healthy skin from surgical patients were transferred to a tube with sterile saline at 4 °C. All patients signed informed consent forms with full knowledge and donated their skin samples voluntarily. Prior approval of the local ethics committee was obtained (ChiCTR1800019082). This study was conducted in accordance with the declaration of Helsinki Principles. CD49f-PE, CD117-BV421, CD146-BV510, CD45-APC-Cy7, CD34-PE-Cy7 and CD29-APC were purchased from Becton Dickinson (San Jose, CA, USA). CD24-BV605 was from BioLegend (San Jose, CA, USA), and the TLR7 antibody (rabbit anti-human, sc-16245) was from Santa Cruz (Santa Cruz, CA, USA); it was used followed by donkey anti-rabbit IgG secondary antibody-Alexa Fluor 488 (Cat number: A-21206 Invitrogen, USA). Each antibody was titrated by serial dilutions. The optimized dilution of all antibodies is listed in Table1. In total, 16 healthy donors (8 males and 8 females) were included in this study with a mean age at 26 (shown in Table 2).

**Table 1:** Final panel of antibodies

	Characteristic being measured	Alternative name	Antibody name	Vendor catalog #	Purpose
			(clone name)	(dilution used)	
BV421	Cell surface protein	c-kit	CD117	BD#562,434	Keratinocytes in rete ridges (Jiang et al. [7])
			(YB5B8)	1:25	
BV510	Cell surface protein	MU138	CD146	BD#563,255	Epidermal appendage (Schon et al. [31])
			(P1H12)	1:100	
BV605	Cell surface protein	Heat-stable antigen(HSA)	CD24	Biolegend#311,123	Differentiated keratinocytes (Thierry Magnaldo [34])
			(ML5)	1:25	
APC	Cell surface protein	Integrin β1	CD29	BD#559,883	Basal cells (Kaur and Li 2000)
			(MAR4)	01:12.5	
APC-Cy7	Cell surface protein	Leukocyte common antigen (LCA)	CD45	BD#557,833	Leukocytes
			(2D1)	1:50	
PE	Cell surface protein	Integrinα6	CD49f	BD#555,736	Epidermal basal cells (Kaur and Li 2000)
			(GoH3)	1:25	
PE-Cy7	Cell surface protein	My10	CD34 (581)	BD#580,710	Outer root sheath of anagen hair follicles cells (Poblet et al. [35])
				1:25	
FITC	Cell surface protein		TLR7	Novus#NBP2-24,906	Basal stem cell (Yin et al. [38])
				1:100	
PI	Double-stranded DNA			Keygen#KGA106	
				1:500

**Table2:** Basic information of the healthy donors for computational analysis

	N = 14	100%	Viability (%)
Sex			
Male	7	50.00	
Female	7	50.00	
Age (years, median ± standard deviation)	28.79 ± 22.81		
Location			
Ear	4	28.57	81.40 ± 8.18
Thorax	5	35.71	80.88 ± 11.79
Abdomen	5	35.71	87.08 ± 5.02

Abdomen includes the abdomen and groin

### Isolation of human keratinocytes

The subcutaneous tissue was cut with scissors, after which the skin sample was washed twice with PBS. The sample was cut into fragments and transferred to 0.25% dispase II (neutral protease, grade II, Cat# 04,942,078,001, Roche, Mannheim, Germany) and incubated for 1.5 h at 37 °C. The epidermis was gently separated with forceps and cut into small pieces. After digestion with 0.125% trypsin (HyClone, Cat No: SH30042.01, GE Healthcare Life Sciences, Sweden) for 8 min at room temperature, the trypsin was neutralized with 20% fetal bovine serum (FBS). The epidermal cells were prepared for staining after filtration with a 70 µm cell strainer, centrifugation at 350*g* for 6 min and discarding of the supernatant.

### Cell staining and flow cytometric analysis

The epidermal cells were resuspended gently, washed with PBS, and then centrifuged at 350*g* for 6 min. After resuspension, antibody cocktail containing 2.0 μl of each antibody (CD49f, CD117, CD146, CD45, TLR7, CD24, CD34 and CD29) was added to 2 million cells with 100 µl of staining buffer. The cell suspension was incubated on ice for 30 min in the dark. The cell suspension was washed twice with PBS, and donkey anti-rabbit IgG secondary antibody-Alexa Fluor 488 (2.0 µl) was added, followed by incubation for 20 min in the dark. After 1.0 µl PI was added for another 5-min incubation, the cell suspension was washed with PBS and centrifuged at 350*g* for 6 min, and the cells were resuspended gently with PBS. The cell suspension was analyzed on a BD FACSAria SORP flow cytometer immediately.

### Data acquisition and analysis pipeline

Acquisition and analysis were performed on a FACSAria SORP cytometer equipped with DivaV6.0 software (Becton Dickinson, San Jose, CA, USA). The instrument setup was standardized to reduce batch-to-batch shifting by daily monitoring with Rainbow beads (Spherotech). The boundary between positive and negative events was placed by fluorescence-minus-one controls. The maximum possible number of events was acquired (at least 500,000 events and preferably more). Data analysis was conducted using Cytobank (Mountain View, CA) and the FlowJo software program (TreeStar, Ashland, OR). In the analysis, a sequential gating strategy was used (Fig. 1). After excluding debris, dead cells and CD45-positive cells, data files of living epidermal cells were concatenated by group and uploaded into Cytobank. ViSNE analyzed 10,000 cells from each sample randomly. The dimensional reduction was visualized on axes identified by tSNE1 and tSNE2. Dimensional reduction and visualization of data files was performed with viSNE (viSNE setting: 10,000/sample, iterations: 7000, perplexity: 50, seed: 94,138,845) followed by SPADE on Cytobank or PhenoGraph (10,000/sample, iterations: 7000, perplexity: 50, seed: 42, k: 45) clustering on R. The SPADE analysis settings were as follows: target number of nodes = 25 and percentage downsampling = 100%. The intensity and cellular abundance of each node from each individual were exported for further analysis. Four categories: high (hi), medium (mi), low (lo), and negative (neg) was divided according to the total expression distribution of cells in each marker. The mean of the median marker expression of the cells contained in each node was then used to assign the expression of each marker to one of the four categories [45]. Statistical data analysis was performed in Prism 8.2.1 (GraphPad Software Inc., La Jolla, CA, USA) and represented as the mean ± SEM. Two-way ANOVA and Student’s t-tests were used to compare data among the ear, thorax and abdomen. A *P *value < 0.05 was considered significant.

## Supplementary Information

**Supplemental Fig.1** Isolation effect of trypsin stained with H&E. (**A**) the skin epidermis structure before trypsinization. The epidermal cells were arranged closely. (**B**) the residual tissue after trypsinization with loose stratum corneum and isolated cells. Star marked the isolated cells. Abbreviations: *SC* stratum corneum; *SB* stratum basal.

## Data Availability

All data generated or analyzed during this study are included in this article.

## Abbreviations

IFE: Interfollicular epidermis
HF: Hair follicle
SB: Stratum basale
SS: Stratum spinosum
SG: Stratum granulosum
SC: Stratum corneum
TLR7: Toll-like receptor 7
