# Supplementary material for: Computational flow cytometric analysis to detect epidermal subpopulations in human skin
Source: Biomed Eng Online. 2021 Feb 17;20:22. doi: 10.1186/s12938-021-00858-8 (PMC7891025; doi:10.1186/s12938-021-00858-8)
Supplement: Supplementary file 1 — Supplemental Fig.1 Isolation effect of trypsin stained with H&E. (A) the skin epidermis structure before trypsinization. The epidermal cells were arranged closely. (B) the residual tissue after trypsinization with loose stratum corneum and isolated cells. Star marked the isolated cells. Abbreviations: SC stratum corneum; SB stratum basal. [file 12938_2021_858_MOESM1_ESM.pdf]

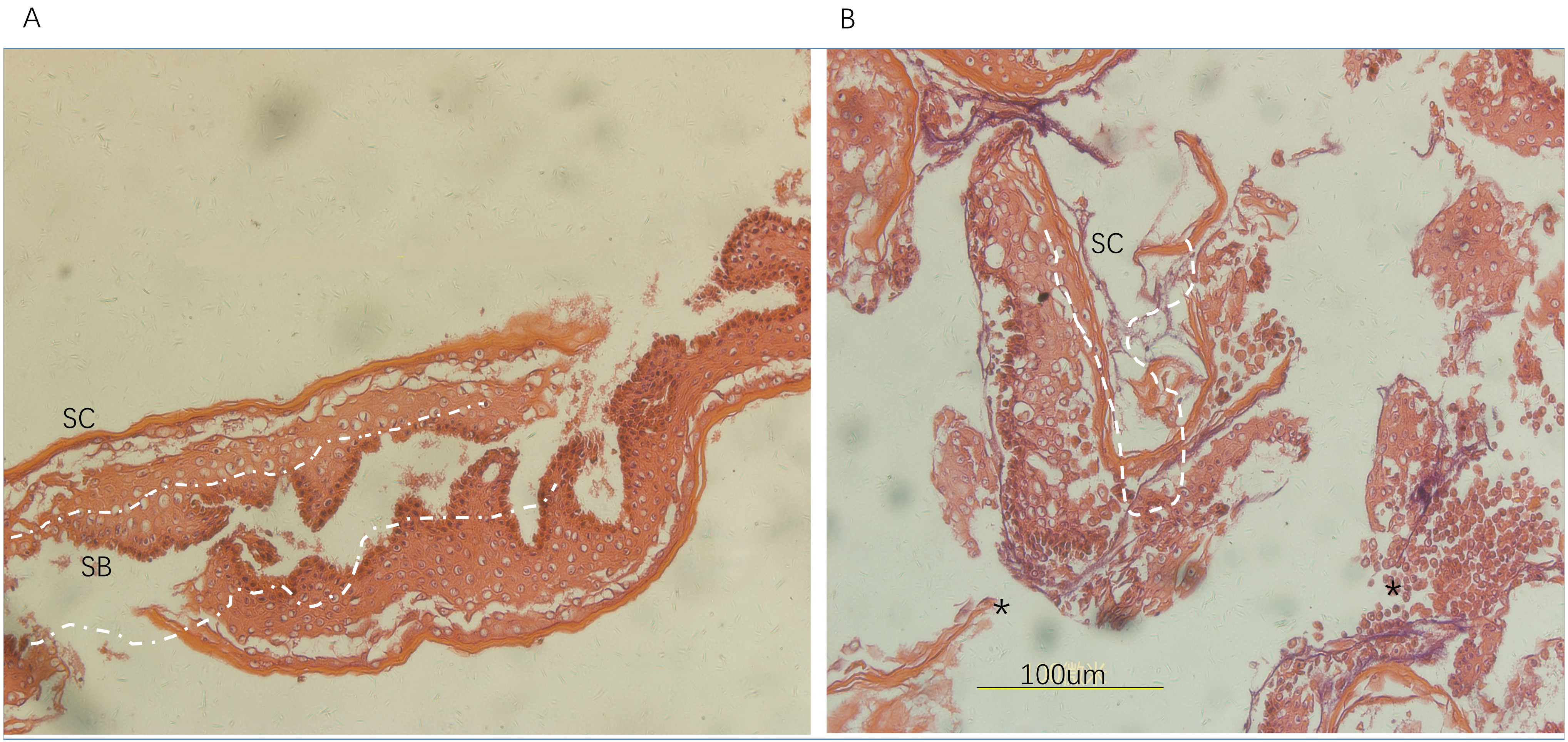

Supplemental Fig.1 Isolation effect of trypsin stained with H&E. (A) the skin epidermis structure before trypsinization. The epidermal cells were arranged closely. (B) the residual tissue after trypsinization with loose stratum corneum and isolated cells. Star marked the isolated cells. Abbreviations: SC, stratum corneum; SB, stratum basal.
